# Evaluation of non‐Gaussian diffusion in cardiac MRI

**DOI:** 10.1002/mrm.26466

**Published:** 2016-09-26

**Authors:** Darryl McClymont, Irvin Teh, Eric Carruth, Jeffrey Omens, Andrew McCulloch, Hannah J. Whittington, Peter Kohl, Vicente Grau, Jürgen E. Schneider

**Affiliations:** ^1^ Division of Cardiovascular Medicine, Radcliffe Department of Medicine University of Oxford Oxford United Kingdom; ^2^ Department of Bioengineering University of California–San Diego La Jolla California USA; ^3^ Department of Medicine University of California–San Diego La Jolla California USA; ^4^ National Heart and Lung Institute Imperial College London London United Kingdom; ^5^ Institute for Experimental Cardiovascular Medicine, University Heart Centre Freiburg, Bad Krozingen, Faculty of Medicine University of Freiburg Freiburg Germany; ^6^ Department of Engineering Science University of Oxford Oxford United Kingdom

**Keywords:** cardiac MRI, diffusion tensor imaging, kurtosis, non‐Gaussian, hypertrophy, tissue characterization

## Abstract

**Purpose:**

The diffusion tensor model assumes Gaussian diffusion and is widely applied in cardiac diffusion MRI. However, diffusion in biological tissue deviates from a Gaussian profile as a result of hindrance and restriction from cell and tissue microstructure, and may be quantified better by non‐Gaussian modeling. The aim of this study was to investigate non‐Gaussian diffusion in healthy and hypertrophic hearts.

**Methods:**

Thirteen rat hearts (five healthy, four sham, four hypertrophic) were imaged ex vivo. Diffusion‐weighted images were acquired at b‐values up to 10,000 s/mm^2^. Models of diffusion were fit to the data and ranked based on the Akaike information criterion.

**Results:**

The diffusion tensor was ranked best at b‐values up to 2000 s/mm^2^ but reflected the signal poorly in the high b‐value regime, in which the best model was a non‐Gaussian “beta distribution” model. Although there was considerable overlap in apparent diffusivities between the healthy, sham, and hypertrophic hearts, diffusion kurtosis and skewness in the hypertrophic hearts were more than 20% higher in the sheetlet and sheetlet‐normal directions.

**Conclusion:**

Non‐Gaussian diffusion models have a higher sensitivity for the detection of hypertrophy compared with the Gaussian model. In particular, diffusion kurtosis may serve as a useful biomarker for characterization of disease and remodeling in the heart. Magn Reson Med 78:1174–1186, 2017. © 2016 International Society for Magnetic Resonance in Medicine.

## INTRODUCTION

Diffusion MRI is used to noninvasively provide information about tissue microstructure 1. Diffusion tensor imaging (DTI) is a widely used method in which a minimum of six diffusion‐weighted (DW) images, in addition to a reference image without diffusion weighting, are used to derive a diffusion tensor 2. In cardiac MRI, the primary, secondary, and tertiary eigenvectors of the diffusion tensor have been shown to correspond to the so‐called fiber, sheetlet, and sheetlet‐normal orientations within the myocardium, respectively 3, 4. Indices derived from DTI such as apparent diffusion coefficient (ADC) and fractional anisotropy (FA) can be linked broadly to a range of tissue properties, including water molecule mobility and coherence of regionally prevailing cell orientation (fiber orientation). In the heart, these indices have been used to assess remodeling of tissue structure in disease states such as hypertrophic cardiomyopathy 5 and after myocardial infarction 6.

The diffusion tensor model assumes that the displacement profile of water diffusion follows a Gaussian distribution. However, it is well established that diffusion in biological tissue deviates from a Gaussian profile as a result of hindrance and restriction from cell and tissue microstructure 7. To address this, a number of models have been proposed to more accurately describe the non‐Gaussian behavior of diffusion in tissue, including the diffusion kurtosis model 8, biexponential models 9, stretched exponential models 10, and statistical models 11, 12, 13. These models have been widely applied in brain MRI, and it has been shown that models of diffusion kurtosis can act as a biomarker for clinical development of Alzheimer's disease 14, provide improved characterization of microstructure in the developing brain 15, and improve characterization of tumors 16 compared with diffusion tensor modeling. Compartmental models of diffusion have also been successful in estimating axon diameter, density, and dispersion 17.

To date, non‐Gaussian analysis of cardiac MRI data has been limited. A bi‐exponential model has been investigated in perfused rat, rabbit, and guinea pig hearts, with the fast and slow components associated with contributions from perfusion and diffusion, respectively 3, 18, 19. It has also been shown that non‐monoexponential diffusion is present in both healthy and infarcted fixed rabbit hearts 20. However, this study was limited to quantifying deviations from the diffusion tensor and did not use models of non‐Gaussian diffusion.

Non‐Gaussian diffusion models may offer metrics that are more sensitive to the presence of restrictions such as cell membranes and organelles. This could be particularly relevant in disease states such as hypertrophy, in which proliferation and enlargement of cardiomyocytes leads to changes in the distribution of cellular restrictions to water diffusion 21. Transverse aortic constriction (TAC) is widely used to surgically generate animal models of heart failure 22. Partial constriction of the transverse aorta via a metal clip or suture results in a rapid increase in left ventricular load with consequent development of hypertrophy, ultimately leading to heart failure.

The aim of this study was to systematically investigate non‐Gaussian diffusion in healthy and hypertrophic fixed rat hearts. A conventional diffusion tensor model as well as a number of non‐Gaussian models were fit to DW images of rat hearts, and ranked based on the Akaike information criterion (AIC) 23. Next, the non‐Gaussian diffusion of healthy hearts was compared with hypertrophic hearts. We hypothesize that non‐Gaussian diffusion modeling provides more sensitive biomarkers in hypertrophic hearts than diffusion tensor modeling. A preliminary version of this work was presented previously 24.

## THEORY

In a standard pulsed‐field‐gradient sequence with rectangular gradient pulses of strength *G* (in T/m), diffusion gradient duration *δ* (in s), and diffusion time *Δ* (in s), the diffusion weighting is quantified by the b‐value, given by $b={\left(\gamma \delta G\right)}^{2}\left(\Delta -\frac{\delta }{3}\right)$ (in s/m^2^), where *γ* is the gyromagnetic ratio (in rad/s/T). Models of diffusion are used to parameterize the relationship between the b‐value and the measured signal attenuation. Assuming a voxel contains a spectrum of diffusion environments, each exhibiting Gaussian diffusion, the measured signal is given by

(1) $$\frac{S\left(b\right)}{S\left(0\right)}=\int^{}P\left(D\right)\;e^{-bD}dD,$$

where P(D) describes the probability density function (PDF) of diffusivity D. Various distributions have been proposed for P(D), including the truncated Gaussian 13, gamma 11, 25, 26, log‐normal 12, 27, and beta 26 distributions. For a given P(D), the mean diffusivity is given by the expectation of D, E[D]. Excess kurtosis (referred to hereafter as kurtosis) is given by the normalized variance of the diffusivity profile as follows:

(2) $$K=3\frac{E\left[D^{2}\right]}{E{\left[D\right]}^{2}}.$$

**Figure 1:**
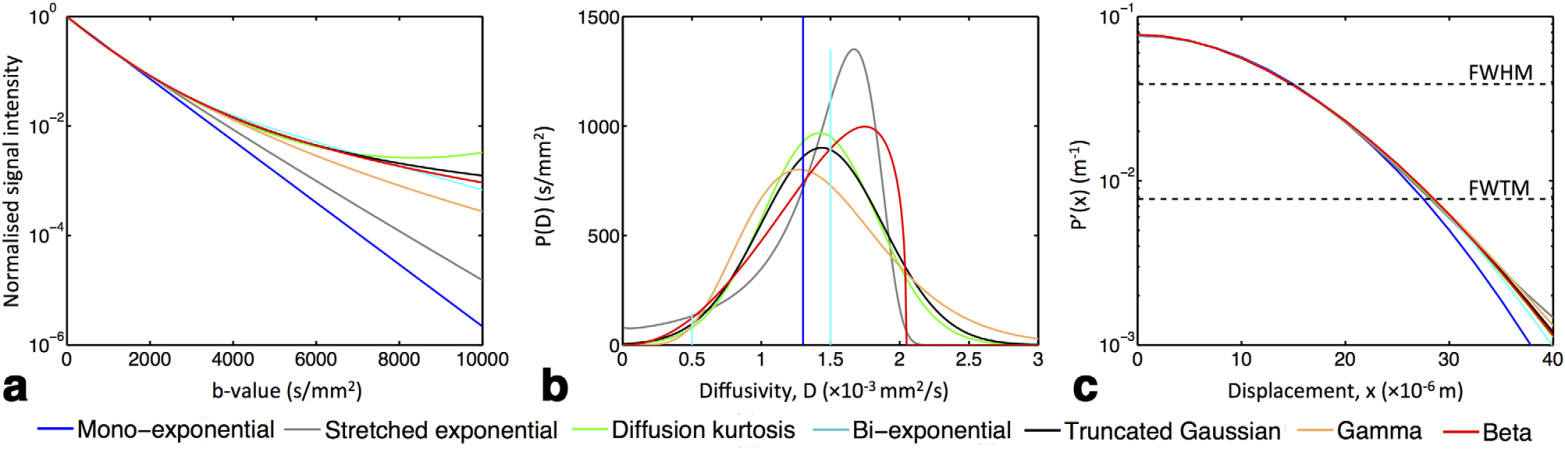
A comparison of model fitting based on synthetic data. All models were fit to data generated using a biexponential model with $D_{\text{fast}}$ = 1.5 × 10^−3^ mm^2^/s, $D_{\text{slow}}$ = 0.5 × 10^−3^ mm^2^/s, and v = 0.9. (**a**) The log‐linear plot of normalized signal intensity versus b‐values shows the similarity of all models for b ≤ 3000 s/mm^2^. (**b**) Diffusivity profile for all models. The relative magnitudes of the fast and slow components of the biexponential model are indicative of their volume fraction. (**c**) Displacement profile for all models. The full‐width half‐maximum (FWHM) and full‐width tenth‐maximum (FWTM) are also displayed. The FWTM gives an estimation of the length scale for the fastest moving molecules 28.

The displacement profile is obtained via the inverse Fourier transform of the signal with respect to the wave vector $q=\frac{\gamma \delta g}{2\pi }$ 28. Figure 1 presents the signal attenuation, diffusivity profile, and displacement profile of selected models. These models are primarily sourced from the review article of Yablonskiy and Sukstanskii 26 and are described in greater detail hereafter. Additional details about mathematical functions required for the models are given in the appendix.

### Monoexponential Model

The well‐known monoexponential model makes the assumption that a voxel contains a single compartment exhibiting unrestricted diffusion. It is derived from the Taylor series of the relationship between the b‐value and logarithm of the MRI signal S(b) as follows:

(3) $$\ln\frac{S\left(b\right)}{S\left(0\right)}=-bD_{\text{app}}+O\left(b^{2}\right).$$

Truncating this series at the first term yields the monoexponential model

(4) $$\frac{S\left(b\right)}{S\left(0\right)}=\text{exp}\left(-bD_{\text{app}}\right),$$

where $D_{\text{app}}$ is the apparent diffusion coefficient (in mm^2^/s). The PDF associated with the monoexponential model is a delta function at $D_{\text{app}}$.

### Stretched Exponential Model

The stretched exponential model was first introduced in 1854 to describe the discharge of a capacitor 29. It was proposed in the context of diffusion MRI by Bennett et al. 10 and later linked to the concept of fractal dimension 30. This model is defined as

(5) $$\frac{S\left(b\right)}{S\left(0\right)}=\text{exp}\left(-b^{a}D_{s}\right),$$

where α is a dimensionless stretching index, 0<a≤1 and $D_{s}$ is the “stretch‐adjusted” diffusivity.

Neither the mean diffusivity nor the diffusion kurtosis can be derived from this model because $b^{\alpha }$ cannot be described by a Taylor series for noninteger values of a. However, a decreased stretching index a may intuitively be interpreted as having greater deviation from a Gaussian displacement profile, which is associated with higher kurtosis. A decreased stretching index has also been associated with more crowded microstructure and more complex water excursions 30. The diffusivity PDF does not have an analytical form but can be computed numerically 31.

### Diffusion Kurtosis Model

The diffusion kurtosis (DK) model 8 is derived from the Taylor series expansion of $\ln\frac{S\left(b\right)}{S\left(0\right)}$ truncated at the second‐order term and is defined as follows:

(6) $$\frac{S\left(b\right)}{S\left(0\right)}=\text{exp}\left(-bD_{\text{DK}}+\frac{K_{\text{DK}}}{6}b^{2}D_{\text{DK}}^{2}\right).$$

The mean diffusivity and kurtosis of this model are given by $D_{\text{DK}}$ and $K_{\text{DK}}$, respectively. The PDF of the DK model is a Gaussian distribution with mean $D_{\text{DK}}$ and variance $\frac{1}{3}D_{\text{DK}}^{2}K_{\text{DK}}$. Thus, kurtosis is a measure of the heterogeneity of the diffusion environment, where higher values of kurtosis indicate a wider spread of apparent diffusivities within a voxel. The decay curve is a quadratic on a log‐scale, with a minimum at $b=\frac{3}{D_{\text{DK}}K_{\text{DK}}}$. The selection of b‐values is important in diffusion kurtosis imaging; the maximum b‐value must be high enough that that the $Ο\left(b^{2}\right)$ is measurable in the presence of noise, but low enough that the truncated $Ο\left(b^{3}\right)$ term is negligible.

### Biexponential Model

The biexponential model 9 assumes that the diffusion signal attenuation can be attributed to two populations, described as a fast and slow component, each of which exhibits Gaussian diffusion.

(7) $$\frac{S\left(b\right)}{S\left(0\right)}=v\;\exp\left(-D_{\text{fast}}b\right)+\left(1-v\right)\text{exp}\left(-D_{\text{slow}}b\right).$$

Parameter v is the volume fraction of the fast component. Mean diffusivity and kurtosis may be computed from the biexponential model as follows:

(8) $$D_{\text{bi}}=vD_{\text{fast}}+\left(1-v\right)D_{\text{slow}}$$

(9) $$K_{\text{bi}}=3\;v\;\left(1-v\right)\frac{{\left(D_{\text{fast}}-D_{\text{slow}}\right)}^{2}}{D_{\text{bi}}^{2}}.$$

The biexponential model corresponds to a PDF consisting of a weighted sum of two delta functions.

### Truncated Gaussian Distribution Model

The truncated Gaussian distribution 13 is appealing because it approximates the DK model at low b‐values but is monotonically decreasing at arbitrarily high b‐values. The PDF is defined as

(10) $$P\left(D\right)=\{\begin{array}{cc} A\exp\left(-\frac{{\left(D-D_{m}\right)}^{2}}{2\sigma^{2}}\right), & D\geq 0\\ 0 & D<0 \end{array},$$

where $D_{m}$ and σ (both in mm^2^/s) control the location and scale of the distribution, respectively, and A is a normalizing coefficient. The normalizing coefficient is dependent on both $D_{m}$ and σ. The observed signal from this model is given by

(11) $$\frac{S\left(b\right)}{S\left(0\right)}=\frac{1+\text{erf}\left(\frac{D_{m}}{\sqrt{2}\sigma }-\frac{b\sigma }{\sqrt{2}}\right)}{1+\text{erf}\left(\frac{D_{m}}{\sqrt{2}\sigma }\right)}\;\text{exp}\left(-bD_{m}+\frac{1}{2}b^{2}\sigma^{2}\right),$$

where erf(·) denotes the error function (see Eq. [A1}). Mean diffusivity, $\bar{D}$, is given by $D_{m}+\sigma \sqrt{\frac{2}{\pi }}\frac{\exp\left(-\frac{D_{m}^{2}}{2\sigma^{2}}\right)}{1+\text{erf}\left(\frac{D_{m}}{\sigma \sqrt{2}}\right)}$, and kurtosis is given by $\frac{3}{{\bar{D}}^{2}}\left(\sigma^{2}-{\bar{D}}^{2}+\bar{D}D_{m}\right)$.

### Gamma Distribution Model

The gamma distribution 11, 25, 26 allows more variation in the shape, and in particular the skewness, than the truncated Gaussian model. The PDF is defined as

(12) $$P\left(D\right)=\{\begin{array}{cc} \frac{1}{\Gamma \left(k\right)\;\theta^{k}}D^{k-1}\;\exp\left(-\frac{D}{\theta }\right), & D\geq 0\\ 0 & D<0 \end{array},$$

where Γ denotes the gamma function (see Eq. [A2]). Parameters k and θ control the shape of the distribution. The observed signal is given by

(13) $$\frac{S\left(b\right)}{S\left(0\right)}={\left(1+b\theta \right)}^{-k}.$$

Mean diffusivity is given by kθ, and kurtosis is given by $\frac{3}{k}$. The skewness of this distribution is given by $\frac{2}{\sqrt{k}}$.

### Beta Distribution Model

A limitation of the truncated Gaussian and gamma distributions is the presence of positive tails with diffusivity greater than that of unimpeded water. The beta distribution model 26 addresses this by imposing an upper limit $D_{\text{max}}$ on the PDF as follows:

(14) $$P\left(D\right)=\begin{array}{cc} \frac{1}{B\left(\alpha ,\beta \right)}{\left(\frac{D}{D_{\text{max}}}\right)}^{\alpha -1}{\left(1-\frac{D}{D_{\text{max}}}\right)}^{\beta -1}, & D\in \left(0,D_{\text{max}}\right) \end{array},$$

where B denotes the beta function (see Eq. [A3]) and parameters α and β control the shape of the distribution. The observed signal is given by

(15) $$\frac{S\left(b\right)}{S\left(0\right)}=M\left(\alpha ,\alpha +\beta ,-bD_{\text{max}}\right),$$

where M is the confluent hypergeometric function (see Eq. [A4]). Because $D_{\text{max}}$ is generally not known, it can be treated as another parameter. Mean diffusivity and kurtosis are given by $\frac{\alpha }{\alpha +\beta }D_{\text{max}}$ and $3\frac{\beta }{\alpha \;\left(\alpha +\beta +1\right)}$, respectively. Skewness is given by $\frac{2\left(\beta -\alpha \right)\sqrt{\alpha +\beta +1}\;}{\left(\alpha +\beta +2\right)\sqrt{\alpha \beta }}$.

## METHODS

### Tissue Preparation

Experimental investigations conformed to the UK Home Office guidance on the Operations of Animals (Scientific Procedures) Act 1986 and were approved by the University of Oxford's ethical review board. Hearts were excised from five healthy Sprague–Dawley rats (body weight = 211 ± 9 g) during terminal anesthesia. Isolated hearts were swiftly perfused in Langendorff constant pressure mode (80 mm Hg) with Krebs‐Henseleit buffer at 37°C (in [mM]: NaCl 118, KCl 4.7, MgSO_4_ · 7H_2_O 1.2, NaHCO_3_ 25, KH_2_PO_4_ 1.2, glucose 11, CaCl_2_ · H_2_O 1.8, and oxygenated with 95% O_2_/5% CO_2_). The hearts were arrested in slack state using high potassium cardioplegic solution (in [mM]: NaCl 125.0, KCl 20.0, MgCl_2_ 1.0, HEPES 5.0, glucose 11.0, CaCl_2_ 1.8; bubbled with oxygen). Time from excision to fixation was approximately 4–5 minutes. The hearts were subsequently perfusion‐ and immersion‐fixed in isosmotic Karnovsky's fixative (300 ± 10 mOsm; in [%]: paraformaldehyde (PFA) 0.45, glutaraldehyde 0.57, sodium cacodylate 0.97) containing 2 mM gadolinium chelate (ProHance; Bracco, Eden Prairie, Minnesota, USA), and stored at 4°C. Prior to imaging, the hearts were rinsed three times in phosphate‐buffered saline with 2 mM gadolinium chelate and embedded in 1% agarose gel (Web Scientific, Crewe, UK) made with phosphate‐buffered saline containing 2 mM gadolinium chelate. Gel was used to retain sample geometric stability, immobility, and hydration, and gadolinium chelate shortened T_1_ and increased signal‐to‐noise ratio (SNR) efficiency.

Eight additional male Sprague–Dawley rats (body weight, 206 ± 5 g) were divided into sham (n = 4) and TAC (n = 4) groups. Animal studies for the sham and TAC groups followed National Institutes Health guidelines and were approved by the University of California San Diego Institutional Animal Care and Use Committee. Animals were anesthetized to an appropriate level with 1.25%–2% inhaled isoflurane, which was confirmed via toe pinch. A lateral thoracotomy exposed the aortic arch. Aortic stenosis was induced in animals in the TAC group by means of a ligature hemo‐clip on the transverse aorta between the right innominate and left carotid arteries with a final, constricted width of 0.5 mm. Sham controls underwent thoracotomy without application of the clip. The surgical site incisions were closed in layers, and the animal was allowed to recover. Four weeks after surgery, M‐mode echocardiographic measurements were made, left ventricular pressures were recorded, and hearts were excised, arrested, fixed, and embedded for imaging in the same manner as the healthy hearts. Imaging was performed approximately 2 wk after fixation.

### Imaging

Diffusion spectrum imaging data were acquired with q‐space sampled on a three‐dimensional (3D) Cartesian grid. A nonselective 3D fast spin echo sequence was used. Imaging was performed on a 9.4T horizontal bore MRI scanner (Agilent, Santa Clara, California, USA) with shielded gradients (max gradient strength = 1 T/m, rise time = 130 μs), and a transmit/receive quadrature‐driven birdcage coil (inner diameter = 20 mm; Rapid Biomedical, Rimpar, Germany).

For the healthy hearts, the following acquisition parameters were used: pulse repetition time = 250 ms; echo time = 15 ms; echo spacing = 4 ms; echo train length = 8; matrix = 100 × 80 × 80; field of view = 20 × 16 × 16 mm; isotropic resolution = 200 μm; number of non‐DW images = 4; number of DW directions = 257; b_max_ = 10,000 s/mm^2^; diffusion duration δ = 5 ms; diffusion time Δ = 9 ms; acquisition time = 14 h, 30 min. The images were acquired at 20.2°C. Systematic temperature bias was minimized by pseudo‐randomizing the DW acquisition such that consecutive scans were acquired with widely different b‐values 32. Diffusion MRI experiments were followed by high‐resolution anatomical imaging using the following parameters: pulse repetition time = 20 ms; echo time = 4 ms; flip angle = 30°; matrix = 600 × 480 × 480; field of view = 20 × 16 × 16 mm; isotropic resolution = 33 μm; acquisition time = 10 h, 14 min. The acquisition protocol for the sham and TAC hearts was identical to that for the healthy hearts, with the following exceptions: matrix = 120 × 80 × 80; field of view = 21.6 × 14.4 × 14.4 mm; isotropic resolution = 180 μm; number of DW directions = 514; acquisition time = 28:46 h. Symmetric q‐space acquisition and higher imaging resolution was used in the latter protocol due to greater availability of scan time.

### Data Analysis

The noise distribution of the magnitude of MR signals with complex Gaussian noise is Rician. The non‐zero mean of the noise results in biased estimates of parameters when fitting using least‐squares minimization. This is particularly apparent in data with low SNR, as is common in high b‐value imaging. To mitigate this bias, the phase was removed, yielding a zero‐mean Gaussian noise distribution 33. Specifically, the mean of the complex non‐DW images was computed, and the phase was subtracted from both the non‐DW and DW images. The real component of the resulting images was extracted. These images can be assumed to have normally distributed noise and therefore can accommodate model fitting using least squares minimization algorithms. The noise level was estimated using the method described previously 34.

The diffusion tensor model was fit to the phase‐corrected data. The eigenvalues of tensor D are given by $\lambda =\left[\lambda_{1}\;\lambda_{2}\;\lambda_{3}\right]$. The mean ADC and FA are defined as

(16) $$\text{Mean ADC}=\lambda =\frac{1}{3}\overset{3}{\underset{i=1}{\sum }}\lambda_{i}$$

(17) $$\text{FA}=\sqrt{\frac{3}{2}\frac{\sum_{i=1}^{3}{\left(\lambda_{i}-\lambda \right)}^{2}}{\sum_{i=1}^{3}\lambda_{i}^{2}}}.$$

Helix, transverse, and sheetlet angle maps were computed from the eigenvectors of the diffusion tensor, using the definitions of angles by Hales et al. 35. The angles were defined relative to a local coordinate system as described by Teh et al. 32 to mitigate biases in angle maps arising from local tissue deformations. The helix angle is the angle subtended by the projection of the primary eigenvector onto the circumferential‐longitudinal plane and the short‐axis plane. The transverse angle is the angle subtended by the projection of the primary eigenvector onto the short‐axis plane and the circumferential‐longitudinal plane. The sheetlet angle is the angle subtended by the projection of the tertiary eigenvector onto the longitudinal‐radial plane and the long axis.

We follow the approach of De Santis et al. 36 in describing the observed attenuation from non‐Gaussian models as the product of the attenuation in the direction of each of the three eigenvectors, as follows:

(18) $$\frac{S\left(b\right)}{S\left(0\right)}=\prod_{i=1}^{3}f\left(b_{i}\right),$$

where $b_{i}$ is the magnitude of the diffusion weighting b in the direction of the *i*th eigenvector of D and f is given by the right‐hand side of Eqs. (4), (5), (6), (7), 11, 13, and 15. This approach assumes that non‐Gaussian diffusion shares the reference frame of the diffusion tensor model. Thus, each of the non‐Gaussian models required six additional parameters to define the reference frame. A list of models in this study and total number of parameters for each is given in Table 1.

**Table 1:** List of Models and Fitting Parameters

Model	Number of Parameters	Parameter List (n)	Initialization Method
DT	7	*S*(0) (1), DT (6)	Linear least‐squares
Stretched exponential	13	*S*(0), DT, **D**_s_ (3), **a** (3),	DT model
DK	13	*S*(0), DT, **D**_DK_ (3), **K**_*DK*_ (3)	Linear least‐squares
Biexponential	14	*S*(0), DT, **D**_fast_ (3), **D**_slow_ (3), *v* (1)	Fitting fast and slow components individually
Truncated Gaussian	13	*S*(0), DT, **D**_*m*_ (3), **σ** (3)	DK model
Gamma	13	*S*(0), DT, **θ** (3), **k** (3)	DK model
Beta	14	*S*(0), DT, **α** (3), **β** (3), *D*_max_ (1)	DK model

All of the non‐Gaussian models require the definition of the DT as the models are fit along the major axes of this tensor. Parameters are described in the Theory section.

Each of the models was fit to all data sets (healthy, sham, and TAC) using nonlinear least squares regression. All models were fit in MATLAB R2013A (MathWorks, Natick, Massachusetts, USA) using a trust‐region‐reflective algorithm 37, with positivity constraints enforced where appropriate. The source code can be obtained through the gerardus project (https://github.com/vigente/gerardus/tree/papers). The initial values of the diffusion tensor and diffusion kurtosis model parameters were obtained using linear least squares regression on the logarithm of the signal. The input data to the DK model was restricted to a maximum b‐value of 5000 s/mm^2^ 25.

Initial values of biexponential model parameters were determined by fitting a monoexponential model to low (<1000 s/mm^2^) and high (>6000 s/mm^2^) b‐values to estimate the fast and slow diffusion rate, respectively 38. The stretched exponential model was initialized with the fit from the diffusion tensor model (i.e., α = 1). In the case of the truncated Gaussian, gamma, and beta distribution models, initial estimates of parameters were derived using the mean diffusivity and kurtosis of the DK model. The parameter controlling the maximum diffusion coefficient in the beta distribution model, $D_{\text{max}}$, was initialized with the diffusion coefficient of pure water at room temperature, 2.3 × 10^−3^ mm^2^/s 39.

### Region of Interest Selection

**Figure 2:**
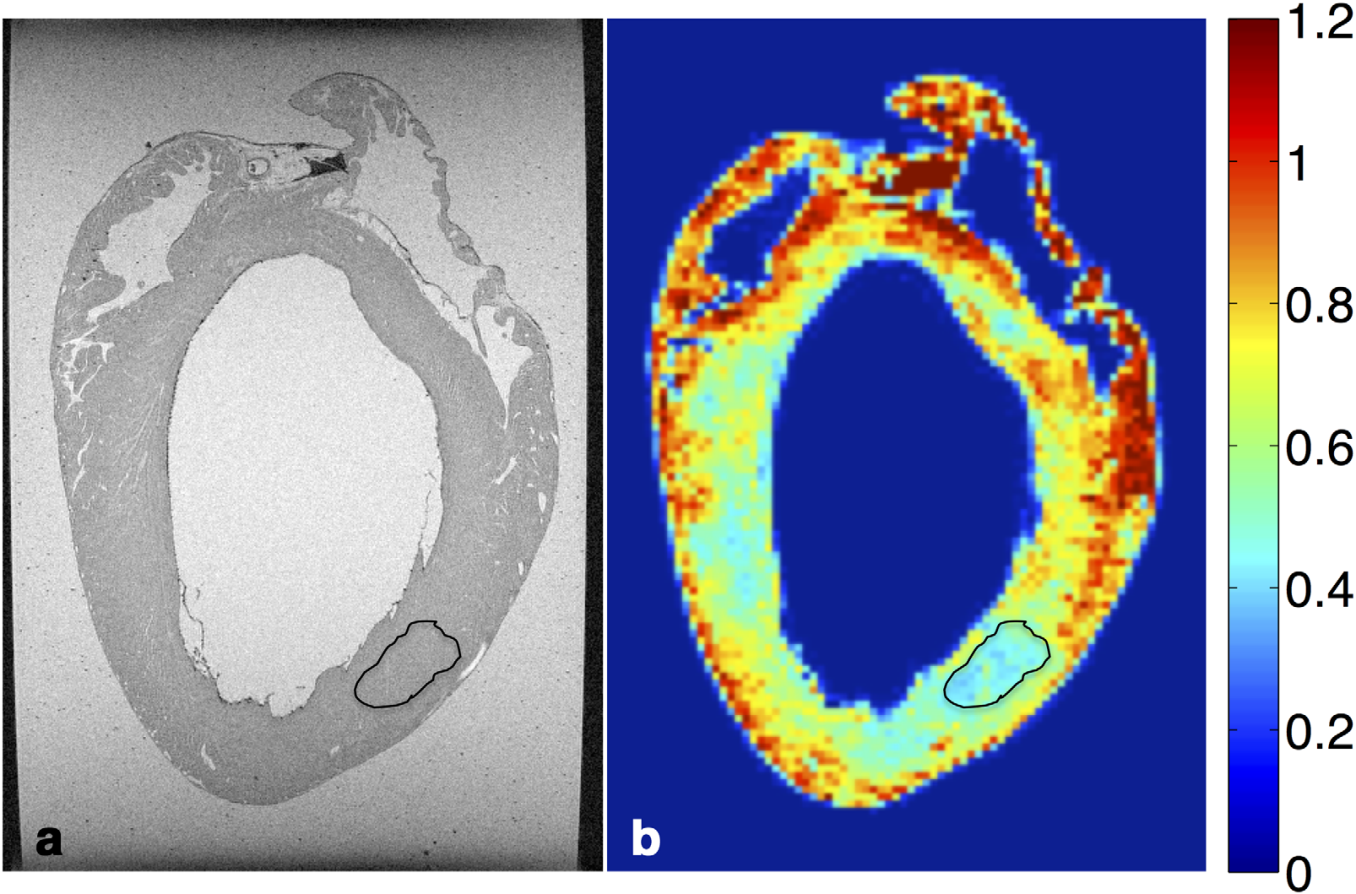
High‐resolution anatomical image (**a**) and kurtosis in the tertiary eigenvector (**b**) in heart 6 (sham). High kurtosis correlates well with regions in the anatomical image that contain buffer. ROIs were traced in hypointense regions on kurtosis maps, with reference to the anatomical images. The ROIs were used for all comparisons between the healthy, sham, and TAC hearts.

A 3D region of interest (ROI) was manually traced on diffusion kurtosis maps in each heart, with reference to high‐resolution anatomical images (Fig. 2a). In each case, the ROI was located in the myocardium of the left ventricle lateral wall. Care was taken to avoid regions containing buffer, gel, or residual blood, as the kurtosis in these regions was artificially increased, as shown in Figure 2b. The ROIs had a volume of at least 1.6 mm^3^ (247 ± 53 voxels).

### Evaluation Using AIC_c_

Complex models having a larger number of fitting parameters provide lower residual errors, but may overfit the data, resulting in high parameter variance in the presence of noise. Conversely, simpler models with fewer parameters return lower parameter variance, but may fail to recover relevant information in the data.

This trade‐off can be quantified using the AIC 23, which is based on information theory. The model selected by the AIC is the one that minimizes information loss. The AIC was selected for model comparison because it provides a quantitative estimate of the amount of information lost by different models and does not require the arbitrary selection of a cutoff.

Performance was compared using the AIC with a correction for finite sample sizes 40, given by

(19) $${\text{AIC}}_{c}=n\log\left(\varepsilon^{2}\right)+2P+\frac{2P\left(P+1\right)}{n-P-1},$$

where $\varepsilon^{2}$ is the mean squared error, *n* is the number of DW images, and *P* is the number of model parameters (including $\varepsilon^{2}$). Thus, P was equal to 8 for the DTI model; 14 for the stretched exponential, diffusion kurtosis, truncated Gaussian, and gamma distribution models; and 15 for the biexponential and beta distribution models. Because AIC_c_ is a relative measure, the difference between the AIC_c_ of each model and the minimum AIC_c_ was recorded. The relative likelihood of model i (i.e. the probability that this model minimises the estimated information loss) is given by

(20) $$p^{i}=\text{exp}\left(\frac{{\text{AIC}}_{c}^{\text{min}}-{\text{AIC}}_{c}^{i}}{2}\right),$$

where ${\text{AIC}}_{c}^{\text{min}}$ refers to the minimum AIC_c_ of the models considered.

To demonstrate b‐value dependence, all models were fit to the data in the ROIs in each of the nine hearts, with diffusion data restricted to b‐values lower than the maximum b‐value. The maximum b‐value was varied between 1200 s/mm^2^ and 10,000 s/mm^2^ in intervals of 400 s/mm^2^. AIC_c_ and relative likelihood was computed in each case. The mean of the relative likelihood over all hearts in each of the three categories (healthy, sham, and TAC) is presented.

## RESULTS

### Pressure Overload

Aortic pressures measured in the TAC group before fixation verified the pressure overload, with the TAC animals having a greater left ventricular peak pressure compared with sham (198 ± 43 mm Hg versus 120 ± 10 mm Hg). This resulted in an increase in heart weight to body weight ratio (4.8 ± 0.2 g/kg versus 3.5 ± 0.2 g/kg). Furthermore, the interventricular septum thickness was higher in TAC versus sham (1.46 ± 0.07 mm versus 1.19 ± 0.02 mm) at end‐diastole but not end‐systole (1.86 ± 0.21 mm versus 1.58 ± 0.09 mm), whereas the internal dimension of the left ventricle was not different between groups at end‐diastole (7.94 ± 0.79 mm versus 7.40 ± 0.32 mm) or end‐systole (4.93 ± 0.53 mm versus 4.18 ± 0.26 mm).

### Gaussian Diffusion Modeling

**Figure 3:**
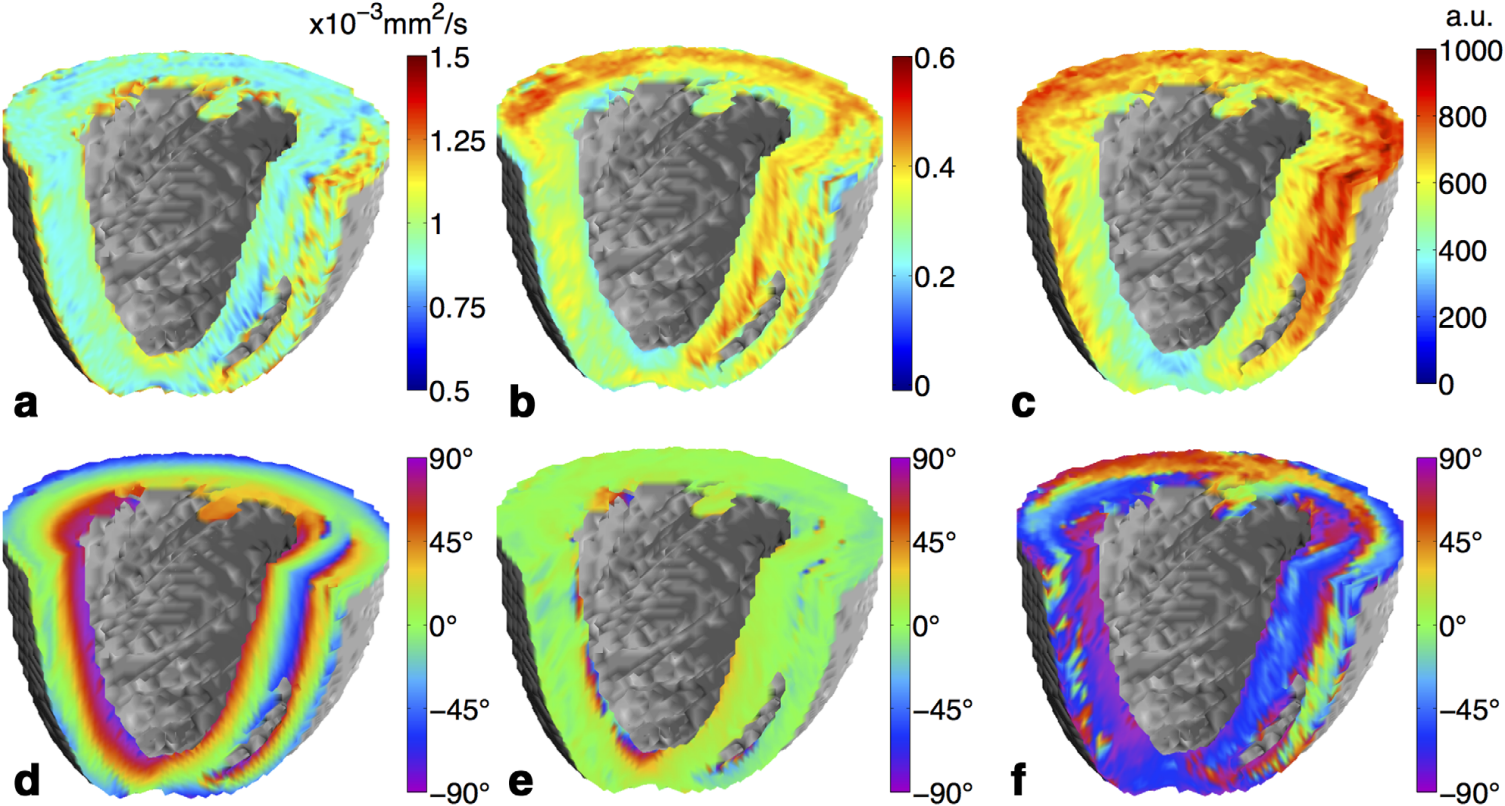
Digitally resected parameter maps derived from Gaussian fitting of a healthy heart. (**a**) Mean apparent diffusion coefficient. (**b**) Fractional anisotropy. (**c**) RMSE. (**d**) Helix angle. (**e**) Transverse angle. (**f**) Sheetlet angle.

Figure 3 presents parameters derived from the Gaussian model of diffusion in a representative healthy heart. The tensor model was fit to data with b‐values up to 2000 s/mm^2^. The mean ADC across the five healthy hearts is 1.01 ± 0.02 × 10^−3^ mm^2^/s in the left ventricle and 1.18 ± 0.04 × 10^−3^ mm^2^/s in the right ventricle. The mean fractional anisotropy is 0.32 ± 0.01 in the left ventricle and 0.25 ± 0.01 in the right ventricle.

Helix angles indicate the well‐known continuous transition between left‐handed and right‐handed cell orientations. Transverse angles close to 0° indicate that the cells have a predominantly circumferential orientation when projected onto the short‐axis plane. Sheetlet angle maps display distinct clusters of voxels with similar sheetlet angles.

**Figure 4:**
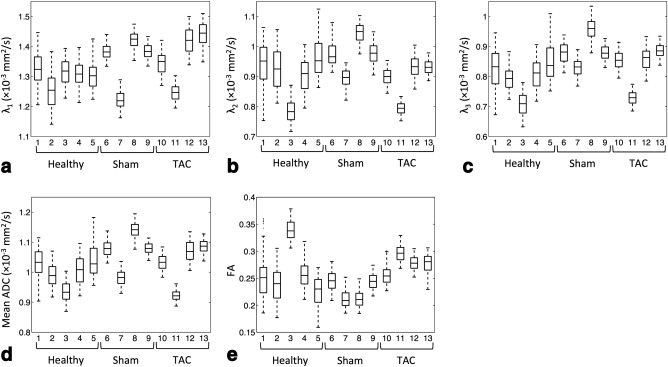
Box‐and‐whisker plots of parameters derived from the diffusion tensor model. Although the mean of the TAC hearts was lower than that of the sham hearts in $\lambda_{2}$ and $\lambda_{3}$, there is considerable overlap, particularly between hearts 7 and 8. As a result, there is poor separation between hearts in terms of mean ADC and FA.

Box‐and‐whisker plots of parameters derived from the diffusion tensor model in the ROIs are presented in Figure 4. On average, the diffusivity in the secondary and tertiary eigenvectors was lower in the TAC hearts compared with the sham hearts, resulting in increased fractional anisotropy. However, there is a large overlap in all DTI‐derived parameters in the healthy, sham, and TAC hearts. The interquartile ranges of the normal hearts are greater than that of the sham and TAC hearts, most likely because of the smaller number of samples in the normal hearts (29 unique samples in q‐space versus 41 in the sham and TAC hearts with b‐values < 2000 s/mm^2^) leading to lower precision.

### Model Selection Using AIC_c_

**Figure 5:**
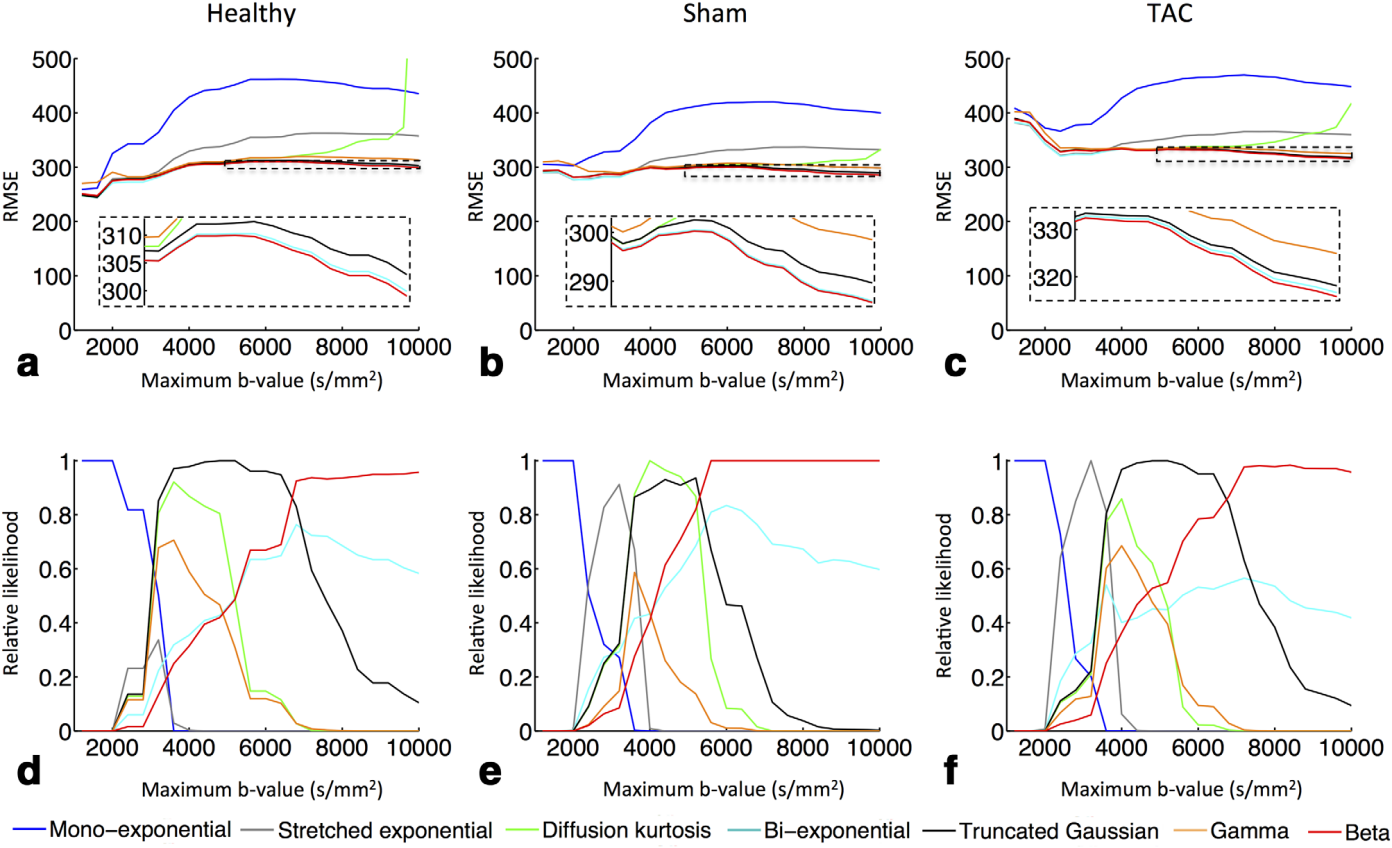
Relationship between maximum b‐value and model performance. (**a**–**c**) RMSE of the healthy (a), sham (b), and TAC (c) hearts for maximum b‐values between 1200 and 10,000 s/mm^2^, averaged over the ROIs. At the higher b‐values, the beta distribution model has the lowest RMSE. The standard deviation of the image noise is 243 ± 17. The mean signal intensity in the non‐DW images is 2.36 ± 0.19 × 10^4^. (**d**–**f**) Relative likelihood of each of the models.

Model selection was highly dependent on the maximum applied b‐value: Figure 5 presents the relationship between model fitting performance and the maximum b‐value employed during fitting. In all hearts, the beta distribution model yielded the model with the lowest root‐mean‐square error (RMSE) and highest relative likelihood at maximum b‐values up to 10,000 s/mm^2^ (i.e., when all data were considered). At maximum b‐values between approximately 3000 s/mm^2^ and 7000 s/mm^2^, the truncated Gaussian model yields the highest relative likelihood in the healthy and TAC hearts, despite having a higher RMSE than the beta or biexponential models. In the sham hearts, the DK model outperformed the truncated Gaussian model for maximum b‐values between 3800 s/mm^2^ and 5400 s/mm^2^, although the difference between the two models is small. The stretched exponential model yielded the highest relative likelihood for maximum b‐values between approximately 3000 and 3800 s/mm^2^ in the sham and TAC hearts. Finally, in all hearts, the conventional diffusion tensor model yielded the highest relative likelihood for maximum b‐values up to approximately 2000 s/mm^2^.

**Figure 6:**
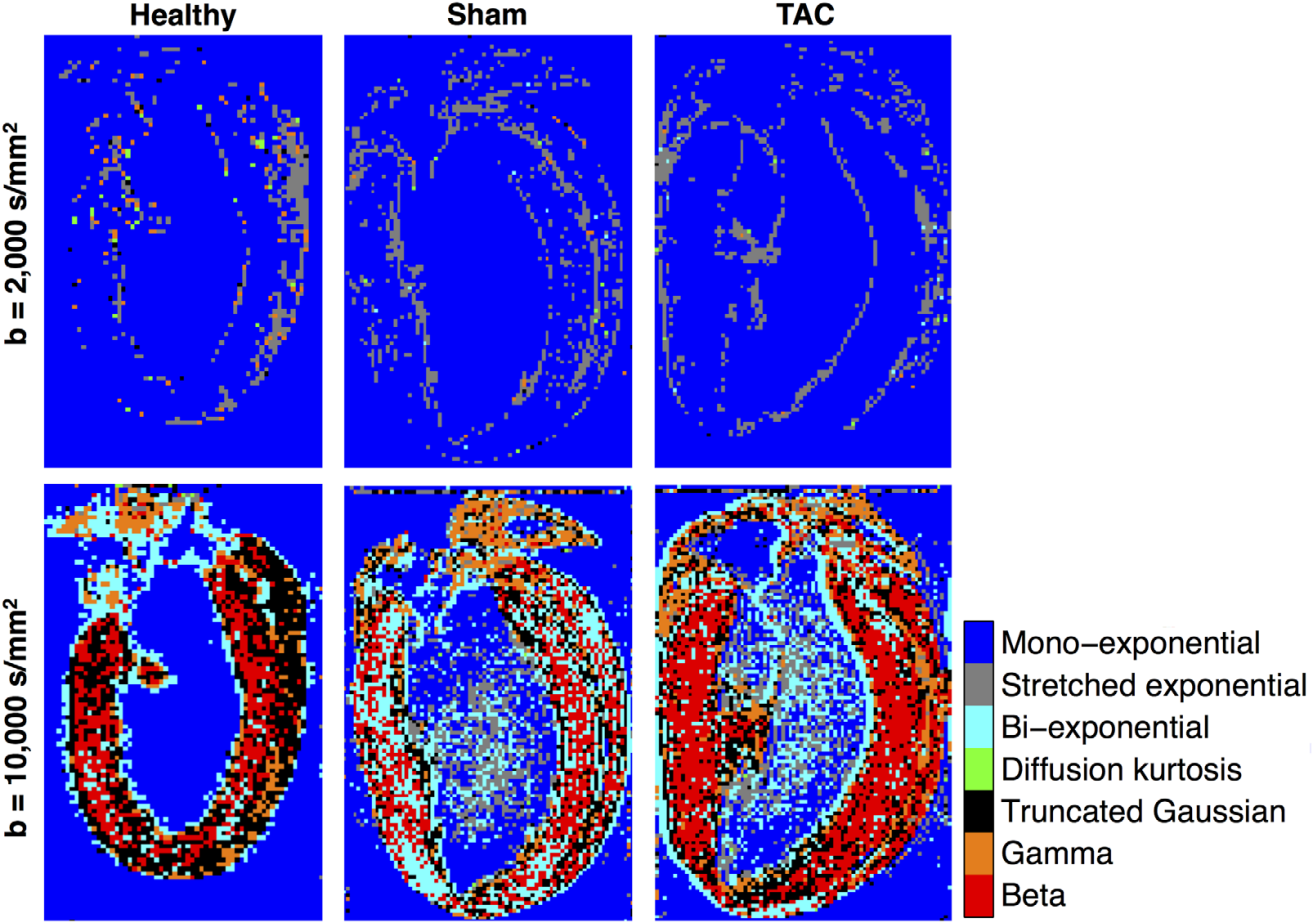
Maps of the model with the highest relative likelihood at maximum b‐values of 2000 (top) and 10,000 (bottom) s/mm^2^.

Figure 6 presents maps of the model with the highest relative likelihood at maximum b‐values of 2000 and 10,000 s/mm^2^. At 2000 s/mm^2^, the diffusion tensor model yields the highest relative likelihood for the majority of voxels in gel, buffer, and the myocardium. The stretched exponential model yields the highest relative likelihood in most voxels on the interfaces between myocardium and gel/buffer. At 10,000 s/mm^2^, the beta model most commonly has the highest relative likelihood in the myocardium. The truncated Gaussian model has the highest relative likelihood in a large number of voxels, particularly in the right ventricle of the healthy heart. The biexponential model has the highest relative likelihood in some myocardial voxels, particularly in the sham heart, and very frequently has the highest relative likelihood on the interfaces between myocardium and gel/buffer. In the voxels on these interfaces, the fast component of the model corresponded to the gel/buffer with diffusivity of approximately 2.3 × 10^−3^ mm^2^/s.

### Kurtosis and Skewness Analysis

**Figure 7:**
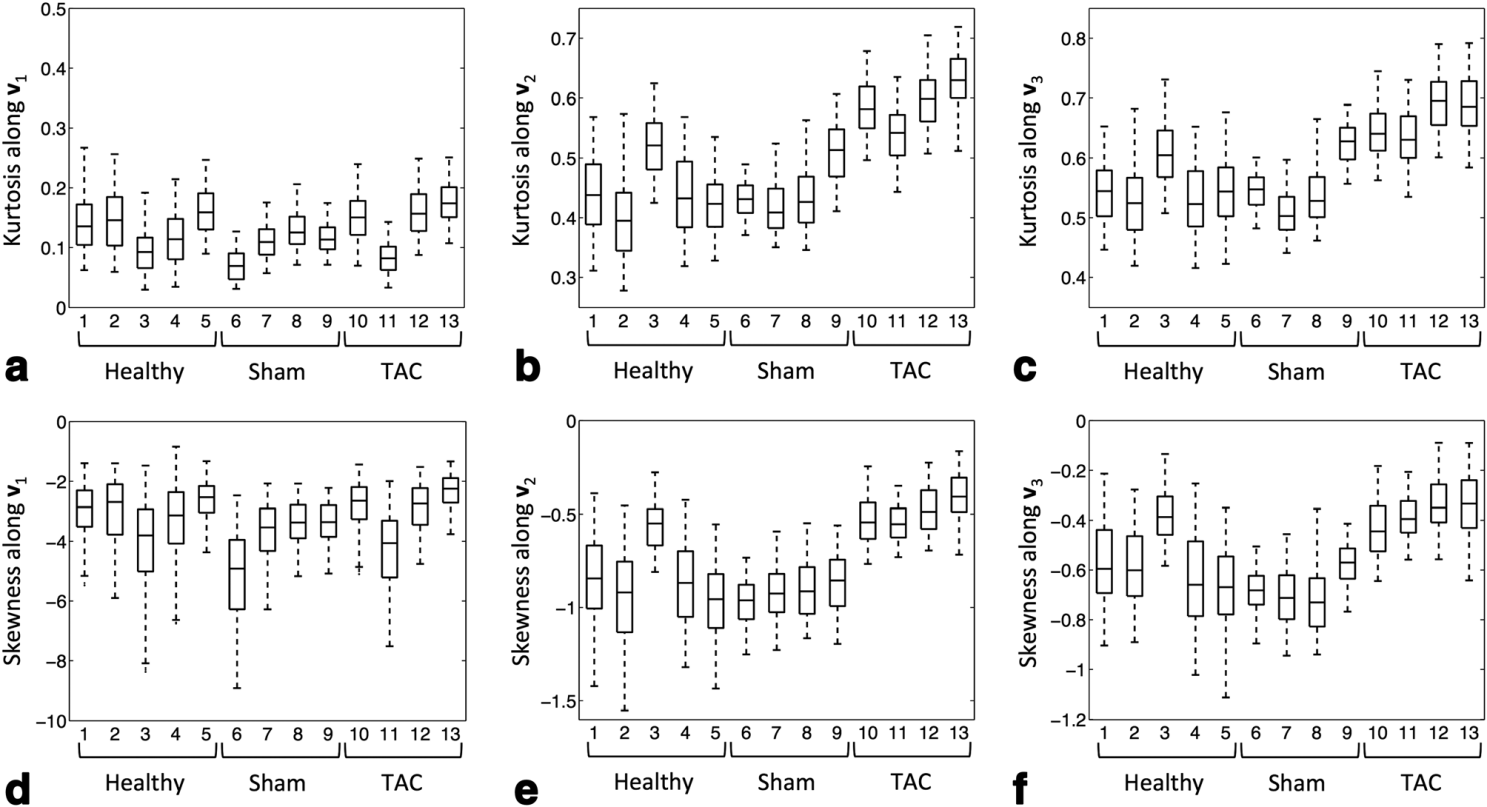
Kurtosis along the principal eigenvectors (**v**_**1**_, **v**_**2**_, and **v**_**3**_) of the diffusion tensor, averaged over the ROIs for healthy, sham, and TAC hearts. Kurtosis was computed from the beta distribution model.

Figure 7 presents box‐and‐whisker plots of kurtosis and skewness generated from the beta distribution model in healthy, sham and TAC hearts, using all b‐values up to 10,000 s/mm^2^. These data are also presented in Table 2. The beta model was selected due to its superior performance when fitting these data, as described above. Both kurtosis and skewness values were lowest in the direction of the primary eigenvector and highest in the direction of the tertiary eigenvector. Skewness was negative in all eigenvector directions, indicating left‐skewed (or left‐tailed) distributions.

**Table 2:** Selected Parameter Values in Healthy, Sham, and TAC Hearts

Parameter	Healthy	Sham	TAC
Biexponential model ***D***_fast_ (10^−3^ mm^2^/s)			
**v**_1_	1.51 ± 0.03	1.52 ± 0.09	1.59 ± 0.12
**v**_2_	1.14 ± 0.07	1.13 ± 0.06	1.08 ± 0.08
**v**_3_	1.04 ± 0.06	1.04 ± 0.05	1.02 ± 0.08
Biexponential model ***D***_slow_ (10^−3^ mm^2^/s)			
**v**_1_	0.48 ± 0.04	0.48 ± 0.06	0.55 ± 0.01
**v**_2_	0.21 ± 0.01	0.19 ± 0.01	0.19 ± 0.01
**v**_3_	0.16 ± 0.01	0.14 ± 0.00	0.17 ± 0.01
Biexponential model *v*	0.86 ± 0.00	0.86 ± 0.01	0.82 ± 0.01
Beta model kurtosis			
**v**_1_	0.13 ± 0.02	0.11 ± 0.02	0.14 ± 0.04
**v**_2_	0.45 ± 0.04	0.45 ± 0.04	0.59 ± 0.03
**v**_3_	0.55 ± 0.03	0.56 ± 0.04	0.67 ± 0.03
Beta model skewness			
**v**_1_	−3.17 ± 0.35	−3.85 ± 0.54	−3.03 ± 0.59
**v**_2_	−0.85 ± 0.15	−0.91 ± 0.04	−0.49 ± 0.05
**v**_3_	−0.57 ± 0.11	−0.67 ± 0.05	−0.37 ± 0.04
Beta model *D*_max_ (10^−3^ mm^2^/s)	1.46 ± 0.04	1.43 ± 0.09	1.50 ± 0.13

Biexponential and beta distribution model parameters (mean ± standard deviation over the ROIs in each of the samples) are given along each of the three eigenvectors.

The sham hearts had similar kurtosis and skewness values to the healthy hearts in all three eigenvectors. Two‐tailed Mann–Whitney *U* tests were performed on the mean of model parameters in each of the ROIs in the sham and TAC hearts, at a 5% significance level. The kurtosis and skewness of the TAC hearts did not differ from that of the healthy or sham hearts in the primary eigenvector, but was significantly higher in the secondary and tertiary eigenvector directions. On average, kurtosis in TAC hearts was 31% higher than in sham hearts in the secondary eigenvector direction (*P* < 0.05), and 20% higher in the tertiary eigenvector direction (*P* < 0.05). Similarly, the skewness of the TAC hearts was 86% higher in the TAC hearts than in the sham hearts in the secondary eigenvector direction (*P* < 0.05), and 81% higher in the tertiary eigenvector direction (*P* < 0.05). Although the mean maximum diffusivity in the TAC hearts was slightly higher (1.50 mm^2^/s) than in the healthy (1.46 mm^2^/s) or sham (1.43 mm^2^/s) hearts, there was a large degree of overlap between the samples in the three categories.

### Biexponential Modeling

Biexponential model parameters are presented in Table 2. The mean volume fraction of fast diffusing components was significantly lower in ROIs in the TAC hearts (0.82 ± 0.01) than in the healthy (0.86 ± 0.00) or sham (0.86 ± 0.01) hearts (*P* < 0.05). On average, the fast diffusing components in the TAC hearts had higher diffusivity in $v_{1}$ and lower diffusivity in $v_{2}$ and $v_{3}$ than the healthy and sham hearts. However, the variance across the hearts in each category was relatively high. Similarly, differences between the TAC and healthy/sham hearts in the slow diffusing component were small relative to the interheart variance.

### Diffusivity PDFs

**Figure 8:**
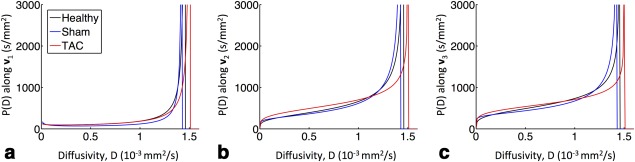
PDFs of diffusivity for the healthy, sham, and TAC hearts derived from the beta distribution model. Profiles were generated from averaged parameters over all ROIs.

Figure 8 presents the PDFs derived from the beta distribution model in the three eigenvectors. These were generated from averaged parameters in the ROIs of healthy, sham, and TAC hearts. The distributions in the primary eigenvector contain sharp peaks at 1.3–1.5 × 10^−3^ mm^2^/s. The distributions in the secondary and tertiary eigenvectors contain peaks at the same location, but with a broader spectrum (i.e., higher kurtosis) and greater contributions from low diffusivities (i.e., more positive skewness).

The diffusivity profiles of TAC hearts in the secondary and tertiary eigenvectors are characterized by broader profiles than the healthy or sham hearts, indicating greater non‐Gaussianity. This is in agreement with the higher volume fraction of the slow diffusion compartment in the biexponential model.

## DISCUSSION

In this study, we investigated the non‐Gaussian behavior of diffusion in ex vivo rat hearts using MRI. Hypertrophic hearts were compared with healthy and sham hearts on the hypothesis that non‐Gaussian parameters will be more sensitive to hypertrophy than parameters derived from the Gaussian model.

In all hearts, greater non‐Gaussianity was observed in the directions of the secondary and tertiary eigenvectors. This may arise from greater restrictions to water diffusion perpendicular to cardiomyocyte orientations. Non‐Gaussianity was highest in the direction of the tertiary eigenvector, which may arise from increased heterogeneity in diffusion environments in the sheetlet‐normal direction. In contrast, the profile of diffusivities in the primary eigenvector was narrower, indicating greater coherence of diffusion and a less restricted environment. These results are in agreement with a previous study on ex vivo pig hearts 41. Nguyen et al. 42 found higher ADC in putative fibrotic regions, as identified with ADC or extracellular volume (ECV) thresholds, in hypertrophic cardiomyopathy patients. This contrasts with our finding that ADC was similar in the sham and TAC groups. It is important to note here that Nguyen et al. focused on the clinically observed manifestation of hypertrophic cardiomyopathy in patients rather than TAC‐induced hypertrophy. Furthermore, there are differences in species, tissue viability, contractility, fixation, and pulse sequence. Further investigation of such differences is needed for more direct comparison of data. Likewise, the community would benefit from greater harmonization of methods and reproducibility and validation studies.

Hypertrophic hearts exhibited significantly greater non‐Gaussianity in the sheetlet and sheetlet‐normal directions than healthy hearts. Specifically, an increase in low diffusivities was observed compared with the healthy and sham hearts. This may be caused by meso‐scale tissue remodeling in response to pressure overload, including cardiomyocyte hypertrophy, decreased capillary density, and interstitial fibrosis 43.

The optimal model to describe the data was found to be highly dependent on maximum b‐value. At b‐values up to approximately 2000 s/mm^2^, the diffusion tensor model yielded the highest relative likelihood. As the maximum b‐value increased, the effects of restricted diffusion are increasingly affecting measured data, and the truncated $Ο\left(b^{2}\right)$ terms begin to become nonnegligible. Thus, the non‐Gaussian models start to fit the data better. At the highest b‐value considered in this study (10,000 s/mm^2^), the beta distribution model offered the best fit, with slightly lower RMSE values than the biexponential model. Although we do not suggest that the distribution of diffusivities necessarily matches either of the distributions suggested by the models—it is clear from Figure 1 that vastly different distributions can yield almost identical signal attenuation profiles—we note that the general trends of these models are in good agreement. Neither model suggests that the maximum diffusivity in the TAC hearts was lower than in healthy or sham hearts, but rather that there was a greater volume fraction of low diffusivities perpendicular to locally prevailing cardiomyocyte orientations. The biexponential model was frequently the best model in regions exhibiting partial volume effects, most likely because this model has the flexibility to represent two distinct populations of diffusion. Although the diffusion kurtosis model frequently did not yield the highest relative likelihood, it was useful for providing initial values for other models, because it can be fit using linear least squares regression. Overall, the truncated Gaussian model offered the best fit out of the 13‐parameter models.

In this study, models of non‐Gaussian diffusion were fit in a diagonalized reference frame, based on the assumption that the principal directions of non‐Gaussian diffusion are the same as those derived from the DTI model. Fitting in the diagonalized reference frame was necessary to make use of statistical models, which are only defined in a single dimension. Although our preliminary work 24 supports the assumption that Gaussian and non‐Gaussian diffusion are well aligned in the myocardium, this removes the possibility to derive the kurtosis tensor 8 and therefore to estimate kurtosis in arbitrary orientations. It also may limit our understanding of the non‐Gaussian behavior of regions with multiple populations of differently oriented cells, such as at the right ventricular wall or papillary muscle insertion points.

In this study, gadolinium chelate was used to reduce tissue T_1_ and scan times. Gadolinium chelate is a known extracellular contrast agent in vivo, and differential localization in the intra‐ and extracellular compartments may affect the diffusion MRI signal. However, the distribution of gadolinium chelate ex vivo depends on the integrity of cell membranes after fixation. One in vitro study found that the T_1_ of fixed cells doped with gadolinium chelate was reduced by >20 × relative to fresh cells doped with gadolinium chelate and suggested that this finding constituted evidence of structural and functional alterations to the cell and nuclear membranes with formaldehyde fixation 44. Another study reported that the transmembrane water exchange rate was dramatically increased after fixation with Karnovsky's fixative 45, as we have used. These suggest that gadolinium chelate may have diffused into the intracellular space, thereby shortening T_1_ in both intra‐ and extracellular compartments. It is also possible that with sufficiently high water exchange rates and long diffusion times, the intracellular and extracellular water may become well mixed. Further investigation would be needed for verification.

At present, it is technically challenging to measure non‐Gaussian diffusion in the heart in vivo. In clinical diffusion imaging, non‐Gaussian models have the potential to help in assessing myocardial perfusion 19. However, gradient hardware in clinical scanners typically limit b‐values to 500 s/mm^2^ 46, 47, 48, reducing sensitivity to non‐Gaussian diffusion arising from finer tissue structures as demonstrated here. Stimulated echo approaches afford higher b‐values for a given maximum gradient amplitude due to long diffusion times. Longer Δ would increase the interactions of water molecules with cellular restrictions and likely enhance non‐Gaussian effects, particularly in the directions of the second and third eigenvectors. However, stimulated echo sequences are subject to myocardial strain, necessitating strain correction 48 or limiting imaging to temporal “sweet spots” 49. Higher b‐values result in lower signal, thus higher SNR is needed to avoid noise floor bias. Furthermore, one DW readout is typically acquired at every other cardiac cycle, and a large number of b‐values are required for non‐Gaussian parameter estimation, increasing acquisition time. These drive the need for better SNR efficiency. Ongoing developments in high performance gradient systems, and the use of spin echo methods 50, convex optimized diffusion encoding 51, simultaneous multislice imaging 52, and compressed sensing 34 promise to improve SNR efficiency and therefore feasibility of in vivo cardiac non‐Gaussian diffusion imaging.

## CONCLUSION

This study represents the first systematic study of non‐Gaussian diffusion in cardiac tissue. Several models of diffusion were presented and applied to fixed ex vivo rat hearts. At b‐values up to approximately 2000 s/mm^2^, the diffusion tensor model yielded the highest relative likelihood, but reflects the signal poorly in the high b‐value regime. The best model at high b‐values, as selected by the AIC, was the beta distribution model. Differences in kurtosis and skewness, but not in DTI‐derived parameters, were observed between healthy and hypertrophic hearts, demonstrating the higher sensitivity of the beta distribution model to pathology compared with the diffusion tensor model. We conclude that non‐Gaussian models best represent the diffusion MRI signal in the myocardium at b‐values exceeding 2000 s/mm^2^, and that parameters derived from these models may serve as useful biomarkers for assessing cardiac disease and remodeling.

## Appendix

Here we provide additional details about a number of mathematical functions described in the Theory section.

The error function is related to the integral of the Gaussian distribution, and is defined as follows:

(A1) $$\text{erf}\left(x\right)=\frac{2}{\sqrt{\pi }}\int_{0}^{x}e^{-t^{2}}dt.$$

The gamma function is an extension of the factorial function from positive integers to real and complex numbers. It is also known as the Euler integral of the second kind and is given as follows:

(A2) $$\Gamma \left(t\right)=\int_{0}^{\infty }x^{t-1}e^{-x}dx.$$

The beta function is the Euler integral of the first kind and is given by

(A3) $$B\left(x,y\right)=\int_{0}^{1}t^{x-1}\;{\left(1-t\right)}^{y-1}dt=\frac{\Gamma \left(x\right)\Gamma \left(y\right)}{\Gamma \left(x+y\right)}.$$

Kummer's function is a standard form of confluent hypergeometric function and is given by

(A4) $$M\left(a,b,z\right)=\sum_{n=0}^{\infty }\frac{a^{\left(n\right)}z^{n}}{b^{\left(n\right)}n!},$$

where $a^{\left(0\right)}=1$, $a^{\left(n\right)}=a\left(a+1\right)\left(a+2\right)\;\cdots \left(a+n-1\right)$ denotes the rising factorial.
